# Essential Oil Derived From *Eupatorium adenophorum* Spreng. Mediates Anticancer Effect by Inhibiting STAT3 and AKT Activation to Induce Apoptosis in Hepatocellular Carcinoma

**DOI:** 10.3389/fphar.2018.00483

**Published:** 2018-05-15

**Authors:** Hao Chen, Bei Zhou, Jie Yang, Xinhua Ma, Shihao Deng, Yun Huang, Yanzhang Wen, Jingquan Yuan, Xinzhou Yang

**Affiliations:** ^1^College of Pharmacy, Guangxi University of Chinese Medicine, Nanning, China; ^2^School of Pharmaceutical Sciences, South-Central University for Nationalities, Wuhan, China; ^3^Guangxi Botanical Garden of Medicinal Plants, Nanning, China

**Keywords:** *Eupatorium adenophorum*, essential oil, anticancer, hepatocellular carcinoma, apoptosis

## Abstract

*Eupatorium adenophorum* Spreng. (EA) is a well-known noxious invasive species. Gas chromatography-mass spectrometry (GC-MS) analysis revealed that the essential oil derived from EA (EAEO) is mainly composed of sesquiterpenes. However, the pharmacological value of EAEO in hepatocellular carcinoma (HCC) remains largely unexplored. Herein, we investigated the anti-HCC activities of EAEO, and explored the potential mechanisms of EAEO-induced apoptosis. An MTT assay showed that EAEO inhibited HCC cell proliferation with little toxicity on normal liver cells. Wound healing and FACS assays revealed that EAEO suppressed HCC cell migration and arrested cell cycle, respectively. Moreover, EAEO promoted *in vitro* HCC cell apoptosis, and EAEO treatment inhibited HepG2 xenografts growth and enhanced apoptotic nucleus of xenografts in HepG2-bearing nude mice. Mechanistically, EAEO significantly decreased the ratio of Bcl-2/Bax and resulted in the activation of caspase-9 and -3. EAEO also reduced the expression of Grp78, which in turn relieved the inhibition of caspase-12 and -7. Meanwhile, EAEO suppressed the phosphorylation of STAT3 and AKT, indicative of its anti-HCC potential. In summary, we determined that EAEO treatment promoted HCC apoptosis via activation of the apoptotic signaling pathway in mitochondria and endoplasmic reticulum, as well as repressed the activity of STAT3 and AKT in HCC cells.

## Introduction

Recent epidemiological trends indicate that the incidence of cancer cases will increase from 14.1 million in 2012 to about 25 million in 2035 (Stewart and Wild, 2014). Hepatocellular carcinoma (HCC), the sixth most common cancer, has the second highest mortality rate of global malignancies. About 780,000 new cases of HCC are diagnosed yearly and over 745,000 deaths are estimated to occur due to HCC (Torre et al., 2015). Only a fraction of HCC patients is eligible for potentially curative treatments, including liver resection, transplantation, and local ablation. Systemic chemotherapy therapy is the most common management strategy for the majority of HCC patients, especially those with late-stage disease (Bruix et al., 2016). However, poor efficacy, adverse drug reactions, and drug resistance are the biggest drawbacks to systemic chemotherapy therapy of HCC. To address these challenges, the identification of efficacious compounds as new potential chemotherapeutic agents against HCC is necessary.

Natural products originating from plants remain the basic source of highly efficient and hypotoxic compounds for anticancer agents. Many chemotherapy agents such as camptothecin, taxol, and doxorubicin, which were derived from natural products, have been successfully applied in clinic extensively. Furthermore, multitudes of anticancer agents researched from the natural products are undergoing clinical evaluations. Hence, there is feasibility in exploring new compounds to fight HCC from natural resources that may meet the growth demands of chemotherapy agent development.

*Eupatorium adenophorum* Spreng. (EA) is a well-known noxious invasive weed belonging to genus Eupatorium. However, EA is traditionally used as a conventional medicine in treating fever, desinsectization, traumatism, and phyma in China (Yunnan Institute of Materia Medica, 1975). Admittedly, translation of EA into available medicinal resources delivers the dual benefit of environmental protection and medicinal purposes. Many plants of genus Eupatorium are used worldwide as conventional medicine: for example, *E. triplinerve* Vahl. is used as folk medicine in India for relieving colic and stomach pain (Selvamangai and Bhaskar, 2012), while *E. fortunei* Turcz. and *E. lindleyanum* DC. are traditional Chinese medicines known to treat respiratory and gynecological diseases for centuries. Thus far, parts of compounds isolated from genus Eupatoriums, such as luteolin, nepetin, and eupatoriopicrin have been shown to treat several knotty diseases as well as cancer (Kupchan et al., 1969; Militao et al., 2004; Suksamrarn et al., 2004). The main compounds in EA have been illuminated and contain a variety of secondary metabolites belonging to flavonoids and sesquiterpenoids (Ding and Ding, 1999). Furthermore, a number of preliminary research studies showed that EA exhibits high phytotoxicity in flora and fauna, and has potential advantages in pest control and bioherbicides (Baruah et al., 1994; Nong et al., 2017). Recently, it was reported that many essential oils contained the major constituent of EA, such as torreyol, aristolone, and α-bisabolol, and exhibited the significant antiproliferative effect on carcinoma (Federica et al., 2009; Khadir et al., 2016; Sobeh et al., 2016). However, few pharmacological studies investigating the anticancer activity of EA have been reported so far. Herein, we aimed to investigate the unexplored value of EA in HCC.

In this study, we prepared the essential oil from the whole plant of *E. adenophorum* (EAEO) and elucidated the main principles of EAEO by gas chromatographic-mass spectrometric (GC-MS) analysis. The further antiproliferative assay of EAEO on HCC cells showed that EAEO inhibited HCC cell proliferation in a dose- and time-dependent manner, but exhibited little cytotoxicity in normal human liver cells. We further unveiled that EAEO had the ability to inhibit HCC cell migration and arrested the cell cycle in G_1_ phase, as well as provoking HCC cells apoptosis. Mechanistically, EAEO-induced HCC cell apoptosis was executed by the activation of apoptosis signaling pathway in mitochondria and endoplasmic reticulum (ER), as well as repressing the activity of STAT3 and AKT.

## Materials and Methods

### Plant Material

The whole plants of EA were collected in Nanning, Guangxi Zhuang Autonomous Region, China in July, 2016 and identified by Professor Jingquan Yuan (Guangxi University of Chinese Medicine, Nanning, China). A voucher specimen (No. SC0184) was deposited in School of Pharmaceutical Sciences, South-Central University for Nationalities, Wuhan, China.

### Preparation of Essential Oil of EA

Air-dried whole plants of EA were mechanically ground and then 5000 g of the powder was used for extracting essential oil by hydrodistillation in a Clevenger-type apparatus in ratio 1:6 of the material to water. EAEO was collected continuously within 12 h, and was dried by anhydrous sodium sulfate. After filtration of anhydrous sodium sulfate, EAEO was collected and stored in a refrigerator at -80°C until needed. The yield of EAEO was 29.5 mL EAEO out of 5000 g EA.

### GC-MS Analysis of EAEO

The volatile constituents in essential oils were separated using an Agilent 7890A gas chromatograph (Agilent Technologies, Palo Alto, CA, United States) with a HP-5MS 5% phenylmethylsiloxane capillary column (30.00 m × 0.25 mm, 0.25 μm film thickness). Helium (99.999%) was used as carrier gas at a flow rate of 2 mL/min, and 0.1 μL samples were injected in split mode distributed in 60:1. Oven temperature was kept at 60°C for 3 min initially, and then raised at the rate of 4°C/min to 260°C. Injector and detector temperatures were, respectively, set at 250 and 280°C. Peak area percents were used for obtaining quantitative data. The gas chromatograph was coupled to an Agilent 5975C mass selective detector (Agilent Technologies, Palo Alto, CA, United States). The EI-MS operating parameters were as follow: ionization voltage, 70 eV; ion source temperature, 200°C. Identification of oil components was accomplished by comparison of their mass spectral fragmentation patterns (WILLEY/ChemStation data system) (Adams, 2007).

### Cell Culture

HepG2, Hep3B, and SMMC-7721 cells are HCC cells originated from human. L02 cells are normal liver cells originated from human. HepG2 and Hep3B purchased from the American Type Culture Collection (Manassas, VA, United States). SMMC-7721 and L02 purchased from Library of Typical Culture of Chinese Academy of Sciences (Shanghai, China). After cells thawing, HepG2, Hep3B, SMMC-7721, and L02 cells were cultured in Dulbecco’s modified Eagle’s medium (DMEM; Sigma-Aldrich, St. Louis, MO, United States) supplemented 10% fetal bovine serum and 1% penicillin/streptomycin (Hyclone, Logan, UT, United States). Above-mentioned cells were maintained in 25 cm^2^ cell culture flasks in a humidified atmosphere containing 5% CO_2_ at 37°C.

### Antiproliferative Activity by MTT Assay

The MTT assay was performed following the protocol (Wilson and Henderson, 2007). HepG2, Hep3B, SMMC-7721, and L02 cells were seeded at 1 × 10^5^ cells/well into 96-well plates and allowed to grow for 24 h. Before treatment, EAEO was dissolved with DMSO as stock solution and diluted by serum-free DMEM to various concentrations (0, 5, 10, 30, 50, 100, 150, and 200 μg/mL). Each dose was set 5 wells. HepG2, Hep3B and SMMC-7721 cells were, respectively, incubated with different concentrations of EAEO for 48 h. After which, the most sensitive cell line and L02 cells were, respectively, incubated with different concentrations of EAEO for 12 and 24 h. Absorbance in each well was measured at 562 nm by a Microplate Reader (Bio-Rad, CA, United States). Calculating the inhibition rate uses the following formula: inhibition rate (%) = [1 - (OD_sample_ - OD_blank_)/(OD_control_ - OD_blank_)] × 100. The IC_50_ value of EAEO is defined as the concentration at which cell inhibition rate was 50% as determined by MTT assays and calculated by the GraphPad Prism 6.0 Software.

### Wound Healing Assay

Wound healing assay was performed as described previously (Liang et al., 2007). HepG2 cells were seeded at 4 × 10^5^ cells/well into 12-well plates and allowed to grow for about 12 h until confluence. After, respectively, incubated with 1.0 mL of EAEO (0, 5, 10, and 20 μg/mL), scratch wounds were photographed immediately as 0 h. And then scratch wound images of the same field were photographed at 12, 24 h after scratch under a phase contrast microscope (Leica, Nussloch, Germany). The measurement of wound surface area was calculated with ImageJ 1.46s software.

### Flow Cytometry Analysis (FACS)

HepG2 cells were seeded at 1 × 10^5^ cells/well into 6-well plates and allowed to grow for 24 h. Cells were, respectively, incubated with 2.0 mL of EAEO (0, 10, 30, and 50 μg/mL) for 24 and incubated with 2.0 mL of EAEO (30 μg/mL) for 12, 24, and 48 h. After cultivation, the cells were preprocessed and performed FACS as the protocol described (Khazaei et al., 2017). Cell cycle distribution was measured by a flow cytometer (BD Biosciences, United States) and analyzed by ModFit LT software.

### Observation of Morphological Changes

HepG2 cells were seeded at 1 × 10^5^ cells/well into 6-well plates and allowed to grow for 24 h. Cells were, respectively, incubated with 2.0 mL of EAEO (0, 10, 30, and 50 μg/mL) and 2.0 mL of CDDP (10 μg/mL) for 24 h. After cultivation, the cellular morphological changes were observed and photographed at a magnification of 80× under a phase contrast microscope (Leica, Nussloch, Germany). After which, the cells were immobilized and stained by Hoechst 33258 (5 μg/mL). Subsequently, the cellular nuclear morphological changes were observed and photographed at a magnification of 80× under a fluorescence microscope (Leica, Nussloch, Germany).

### Protein Preparation and Analysis

HepG2 cells were seeded at 2 × 10^5^ cells/well into 100 mm × 20 mm cell culture dish and allowed to grow for 24 h. Cells were, respectively, incubated with 6.0 mL of EAEO (0, 10, 30, and 50 μg/mL) and 6.0 mL of CDDP (10 μg/mL) for 24 h. After cultivation, cells were collected and lysed in RIPA containing phenylmethanesulfonyl fluoride and PhosSTOP (Nantong, Jiangsu Province, China). Dissected tumor tissues were carried out as the same protocol. The supernatant containing protein was collected and stored at -80°C until use. The measurement of protein content was performed with a BCA kit (Nantong, Jiangsu Province, China). Equivalent amounts of the protein were separated by electrophoresis on various proportion SDS-PAGE and transferred to polyvinylidene difluoride membranes (Bio-Rad, CA, United States). And then the membranes were blocked by 5% skim milk in tris-buffered saline with tween 20 (0.5%) for 2 h. Subsequently, the membranes were incubated with the primary antibody at 4°C overnight and incubated with the secondary antibody at 37°C for 2 h. The HRP ECL system (Nantong, Jiangsu Province, China) was used to detecting the protein band and the film was scanned and saved.

### HCC-Bearing Nude Mouse Model and *in Vivo* Treatment

HepG2-bearing nude mouse model were established as described previously (Su et al., 2015). Briefly, 200 μL of HepG2 cells (1 × 10^7^ per mouse) were subcutaneously transplanted into the right flank of the nude mice (BALB/c, SPF grade, Female, 16–18 g, 4–5 weeks old). When tumors reached 100 mm^3^, the animals were randomized in four groups (*n* = 5 mice per group) and treated i.p. with EAEO (0, 30, 60, and 120 mg/kg/2 days) for about 3 weeks. Tumor size was measured every 4 days. Three weeks later, mice were sacrificed and transplanted tumors were removed for assessments. All of the protocols involved in our animal experiments were in accordance with the Animal Care and Use Committee of South-Central University for Nationalities (Wuhan, China).

### Terminal Deoxynucleotidyl Transferase dUTP Nick End Labeling (TUNEL)

Xenografts were embedded in paraffin and cut into sections (5 μm) after pretreatment with ice-cold saline and fixed in buffered neutral 10% formalin. The sections were subjected to TUNEL assay by TUNEL assay kit (Roche Biotechnology, Basel, Switzerland) according to the manufacturer’s instructions. After staining and sealing, photographs were taken at the magnification of 200×. Apoptosis index was quantified by the yellow-stained apoptotic nucleus. Ten regions were chosen from the photographs of tumor sections randomly, then blinded and counted by two people. Their mean values were used in statistical analysis. To avoid the discrepancy between two observers, a datum was valid only if the discrepancy between the two observers was less than 10%.

### Immunohistochemical Examinations of Transplanted Tumor Tissues (IHC)

Immunohistochemical examinations were conducted as described previously (Yan et al., 2010). Briefly, after embedding in paraffin, xenografts were cut into sections (5 μm) and incubated with the indicated primary antibodies, and visualized by corresponding secondary antibodies conjugated with horse radish peroxidase. After staining and sealing, photographs were taken at the magnification of 200×.

### Statistical Analysis

All data are shown as mean ± SD from three independent experiments. GraphPad Prism 6.0 software was used for analysis. Statistically differences were analyzed by one-way analysis of variance (ANOVA) and *P*-values < 0.05 were considered significant.

## Results

### Chemical Profiling of EAEO by GC-MS

**FIGURE 1:**
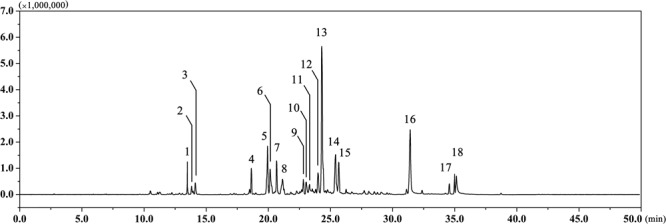
The GC chromatogram of EAEO. The volatile constituents in essential oils were separated using GC chromatogram.

The EAEO was obtained by hydro-distillation with a yield of 0.52% (w/w) according to the dry weight of EA. GC-MS analysis showed that there were 18 different compounds in EAEO, representing 96.33% of the total oil, in which 11 principles belonged to sesquiterpenoids (**Figure 1** and **Table 1**). EAEO was dominantly comprised of torreyol (30.10%), aristolone (11.54%), and α-bisabolol (9.12%), followed by α-curcumene (7.88%), palmitic acid (5.15%), β-bisabolene (4.84%), and β-sesquiphellandrene (4.76%). Among other compounds, a variety of compounds were found at considerable amounts, including 8-cedren-13-ol (4.34%), α-bergamotene (3.56%), β-cedrene (3.26%), caryophyllene oxide (2.42%), spathulenol (2.21%), carvacrol (1.86%), linoleic acid (1.43), thunbergene (1.09%), phytol (0.95%), thymol (0.94%) and anthemol (0.88%).

**Table 1:** Chemical composition (%) of EAEO analyzed by GC-MS.

Number	Name	Ret. Time (s)	Formula	Relative abundance (%)
(1)	Anthemol	16.700	C_10_H_16_O	0.88
(2)	Thymol	16.868	C_10_H_14_O	0.94
(3)	Carvacrol	17.017	C_10_H_14_O	1.86
(4)	α-bergamotene	19.275	C_15_H_24_	3.56
(5)	α-curcumene	19.926	C_15_H_22_	7.88
(6)	β-sesquiphellandrene	20.029	C_15_H_24_	4.76
(7)	β-bisabolene	20.293	C_15_H_24_	4.84
(8)	β-cedrene	20.533	C_15_H_24_	3.26
(9)	Spathulenol	21.370	C_15_H_24_O	2.21
(10)	Caryophyllene oxide	21.481	C_15_H_24_O	2.42
(11)	Thunbergene	21.611	C_20_H_32_	1.09
(12)	8-cedren-13-ol	21.955	C_15_H_24_O	4.34
(13)	Torreyol	22.116	C_15_H_26_O	30.10
(14)	α-bisabolol	22.661	C_15_H_26_O	9.12
(15)	Palmitic acid	22.794	C_16_H_32_O_2_	5.15
(16)	Aristolone	25.676	C_15_H_22_O	11.54
(17)	Phytol	27.246	C_20_H_40_O	0.95
(18)	Linoleic acid	27.472	C_18_H_32_O_2_	1.43

### EAEO Treatment Inhibits HCC Cells Proliferation

**FIGURE 2:**
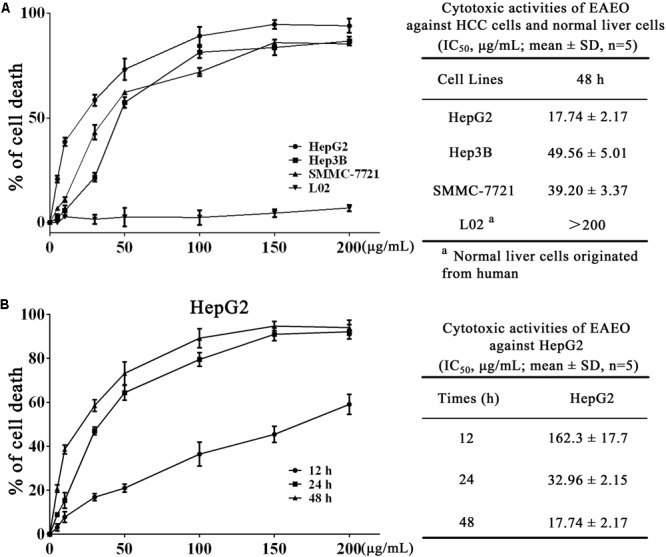
EAEO treatment inhibits HCC cells proliferation. **(A)** HepG2, Hep3B, and SMMC-7721 cells were incubated with 0, 5, 10, 30, 50, 100, 150, and 200 mg/mL of EAEO for 48 h. **(B)** HepG2 cells were incubated with 0, 5, 10, 30, 50, 100, 150, and 200 mg/mL of EAEO for 12, 24, and 48 h. Above inhibition rates and IC_50_ values were determined by MTT assay.

We investigated the antiproliferation of EAEO in HepG2, Hep3B, and SMMC-7721 cells using MTT assay. Those cells were respectively incubated with 0, 5, 10, 30, 50, 100, 150, and 200 μg/mL of EAEO for 48 h. L02 cells, a non-cancerous liver cell line originated from human, was also incubated at the same conditions as HCC cells for evaluating the toxic impacts. Results showed that EAEO dose-dependently enhanced inhibition rates of HepG2, Hep3B, and SMMC-7721 cells. The IC_50_ values of HepG2, Hep3B, and SMMC-7721 cells at 48 h were 17.74 ± 1.92, 49.56 ± 5.01, and 39.20 ± 3.37 μg/mL, but no distinct antiproliferative activity against L02 was observed. The IC_50_ value of L02 was more than 200 μg/mL (**Figure 2A**). Among the three cell lines, HepG2 as the most sensitive HCC cell line was selected to incubate with EAEO for 12, 24, and 48 h. Further studies showed that EAEO dose- and time-dependently enhanced inhibition rate of HepG2 cells. The IC_50_ values of EAEO at 12 and 24 h were 162.3 ± 18.4 and 32.96 ± 2.15 (**Figure 2B**). Basing the above results, HepG2 was selected as the representative HCC cell line and the concentration gradient (0, 10, 30, and 50 μg/mL) of EAEO was pick out for subsequent assay predominantly.

### EAEO Treatment Inhibits HepG2 Cells Migration

**FIGURE 3:**
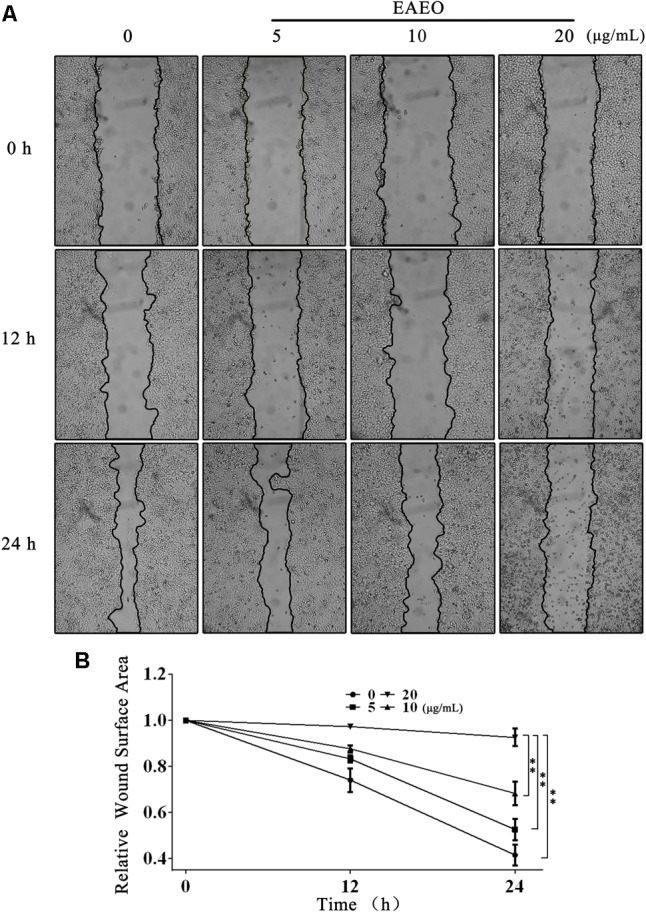
EAEO treatment inhibits HepG2 cells migration. **(A)** HepG2 cells were performed with wound healing assay. After which, the cells were treated with 0, 5, 10, and 20 μg/mL of EAEO and photomicrographed at 0, 12, and 24 h. **(B)** The relative wound surface area was calculated by ImageJ 1.46s. Statistical difference was analyzed by ANOVA, ^∗^*P* < 0.05, ^∗∗^*P* < 0.01 compare to control (0 h served as control).

Collective cell migration is a hallmark of cancer invasion. Quantifying wound healing is a useful assay to evaluate alterations in cancer cell migratory capacity. In our study, wound healing assay was performed to determine the effect of EAEO on HCC cells migration. Because HepG2 proliferation was obviously inhibited when the concentration of EAEO was more than 30 μg/mL, the concentration gradient (0, 5, 10, and 20 μg/mL) of EAEO was pick out for wound healing assay. Photomicrographs showed that the wounds of control treatment cells were significantly healed after 24 h, whereas the wound healing of those receiving EAEO treatment cells were inhibited (**Figure 3A**). Wound surface area analyses showed that EAEO restrained the wound healing in a dose-dependent manner. In addition, the relative wound surface areas of 10 and 20 μg/mL groups were more than 0.6 at 24 h (**Figure 3B**), while the inhibitions on HepG2 proliferation at those concentrations were unconspicuous.

### Effects of EAEO on Cell Cycle in HepG2 Cells

**FIGURE 4:**
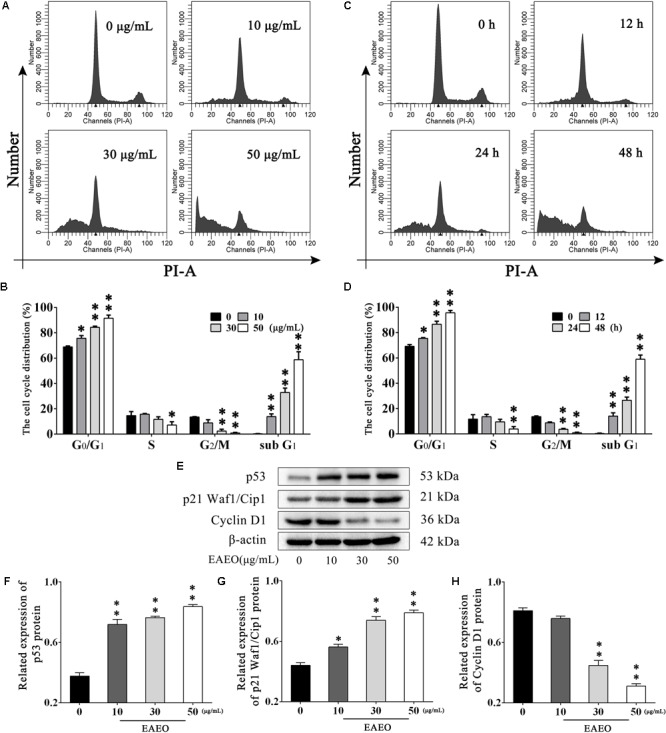
Effects of EAEO on cell cycle in HepG2 cells. **(A)** HepG2 cells were treated with 0, 10, 30, and 50 μg/mL of EAEO for 24 h. After which, the cells were fixed and incubated with PI for 30 min at 4°C followed by FACS. **(B)** The fraction of G_0_/G_1_, S, G_2_/M and sub G_1_ phase in the group treated with concentration gradient. **(C)** HepG2 cells were treated with 30 μg/mL of EAEO for 12, 24, and 48 h. After which, the cells were fixed and incubated with PI for 30 min at 4°C followed by FACS. **(D)** The fraction of G_0_/G_1_, S, G_2_/M, and sub G_1_ phase in the group treated with time gradient. Above fractions were calculated by ModFit LT. **(E)** HepG2 cells were treated with 0, 10, 30, and 50 μg/mL of EAEO for 24 h, after which whole-cells lysates were processed for western blot analysis and probed with the indicated antibodies. **(F–H)** Relative expression of p53, p21 Waf1/Cip1, and Cyclin D1 protein. Statistical difference was analyzed by ANOVA, ^∗^*P* < 0.05, ^∗∗^*P* < 0.01 compare to control (0 μg/mL and 0 h served as control).

To explore the effects of EAEO on HCC cell cycle, flow cytometry analysis (FACS) was performed. HepG2 cells were, respectively, incubated with 0, 10, 30, and 50 μg/mL of EAEO for 24 h. Results showed that EAEO treatment dose-dependently decreased the fraction of G_2_/M phase of HepG2 cells, which was accompanied by a concomitant increase of cells in G_0_/G_1_. However, cells seemed to be inactive in S phase at relatively low EAEO concentrations (10 and 30 μg/mL) (**Figures 4A,B**). Similarly, EAEO also time-dependently decreased the fraction of G_2_/M phase and increased the fraction of G_0_/G_1_ synchronously (**Figures 4C,D**). We were also interested in the effects of EAEO on cell cycle regulatory proteins involved in G_0_/G_1_ arrest (Supplementary Figure S3). Cyclin D1 is an important factor promoting the G_0_/G_1_- to S-phase transition which is critical for the progression of cell cycle; on the contrary, p53 and p21 Waf1/Cip1, are negative regulators of this transition. In all of the HCC cells we tested, EAEO dose-dependently declined protein levels of Cyclin D1, and dose-dependently increased p53 and p21 Waf1/Cip1 (**Figures 4E–H**). These data indicated that EAEO was capable of inducing G_0_/G_1_ arrest, and the blockage effects on cell cycle might prevent HCC cells from entering M phase.

### EAEO Induces HepG2 Cells Apoptosis *in Vitro*

**FIGURE 5:**
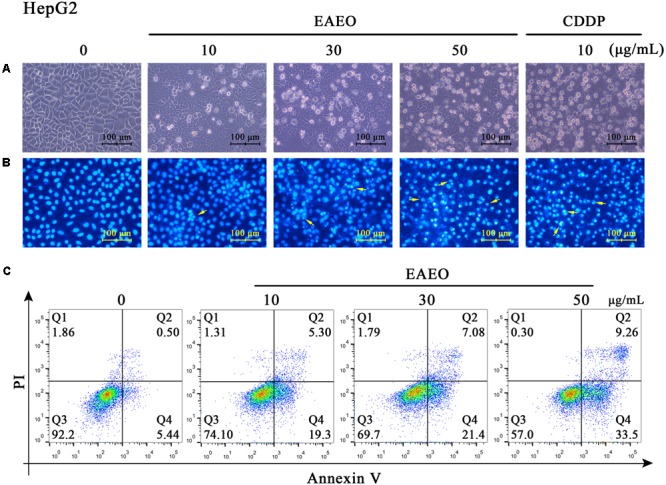
Observation of morphological changes in HepG2 cells. **(A)** HepG2 cells were treated with 0, 10, 30, and 50 μg/mL of EAEO and 10 μg/mL of CDDP for 24 h, after which the cells were photographed under a phase contrast microscope. **(B)** Then, the cells were fixed and incubated with Hoechst 33258 for 30 min, after which the cells were photographed under a fluorescence microscope. CDDP group served as positive control and the yellow points indicated the apoptosis characteristics of cell shape **(C)** HepG2 cells were treated with 0, 10, 30, and 50 μg/mL of EAEO for 24 h. After which, the cells were incubated with PI and Annexin V, respectively, for 10 min at 4°C followed by FACS. Above fractions were calculated by FlowJo 10.

**FIGURE 6:**
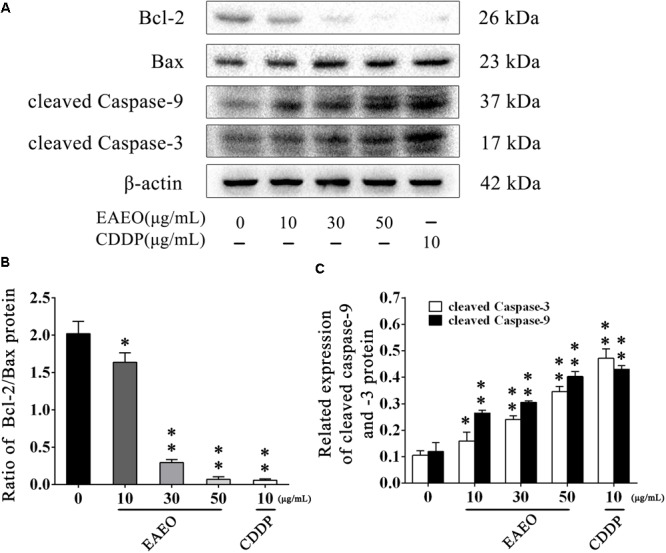
Effects of EAEO on the apoptosis regulatory proteins in HepG2 cells. **(A)** HepG2 cells were treated with 0, 10, 30, and 50 μg/mL of EAEO and 10 μg/mL of CDDP for 24 h, after which whole-cells lysates were processed for western blot analysis and probed with the indicated antibodies. **(B)** Ratio of Bcl-2/Bax protein. **(C)** Relative expression of cleaved caspase-9 and -3 protein. Statistical difference was analyzed by ANOVA, ^∗^*P* < 0.05, ^∗∗^*P* < 0.01 compare to control (0 μg/mL served as control).

Sub G_1_ phase as a representative hallmark indicating apoptosis was dose- and time-dependently increased by EAEO treatment (**Figure 4**). To confirm EAEO-induced apoptosis, we compared the morphological changes of EAEO-treated HCC cells and CDDP-treated HCC cells with positive group. As depicted by the photomicrographs under a phase contrast microscope, we observed the typical apoptosis characteristics of cell shape and labeled them with the yellow points, such as shrinkage, distortion, and flotage with progressing EAEO concentrations (**Figure 5A**). Similar apoptotic changes of nucleus were also observed by Hoechst 33258 staining assay. Photomicrographs indicated that the quantity of chromatin condensation and apoptotic bodies increased in a dose-dependent manner and the effects induced by 50 μg/mL of EAEO were similar to the CDDP-treated group (**Figure 5B**). To further monitor the cell apoptosis, we performed Annexin-V FITC/PI staining and found that the fraction of Q2 and Q4 gradually increased. It indicated that EAEO treatment time-dependently induced early and late apoptosis. We were also interested in exploring the effects of EAEO on the expression of apoptosis regulatory proteins. Bcl-2 family (including Bcl-2 and Bax) regulates intrinsic apoptosis senses of death signals (Fox and MacFarlane, 2016). Previous studies have reported that the balance of Bcl-2/Bax determines the initiation of mitochondrial apoptosis, and a high ratio of Bcl-2/Bax is considered anti-apoptotic (Shi et al., 2017). Caspase-9 and caspase-3 play the executioner role in apoptosis, and upon their activation, their precursor forms are cleaved (Lopez and Tait, 2015). Hence, we investigated the expression of Bcl-2, Bax, cleaved caspase-9 and cleaved caspase-3 using western blot analysis (**Figure 6A**). Decrease of Bcl-2/Bax and increase of cleaved caspase-9 and -3 are typical apoptotic indicators following CDDP treatment, which was observed in our study. Furthermore, EAEO treatment significantly reduced the ratio of Bcl-2/Bax, while increased the expressions of cleaved caspase-9 and -3 in a dose-dependent manner (**Figures 6B,C**). Collectively, these data suggest that EAEO has the potential to induceHCC cell apoptosis in vitro by activation of the mitochondrial apoptotic pathway (Supplementary Figure S1).

### EAEO Inhibits the Tumor Growth and Induces Apoptosis *in Vivo*

**FIGURE 7:**
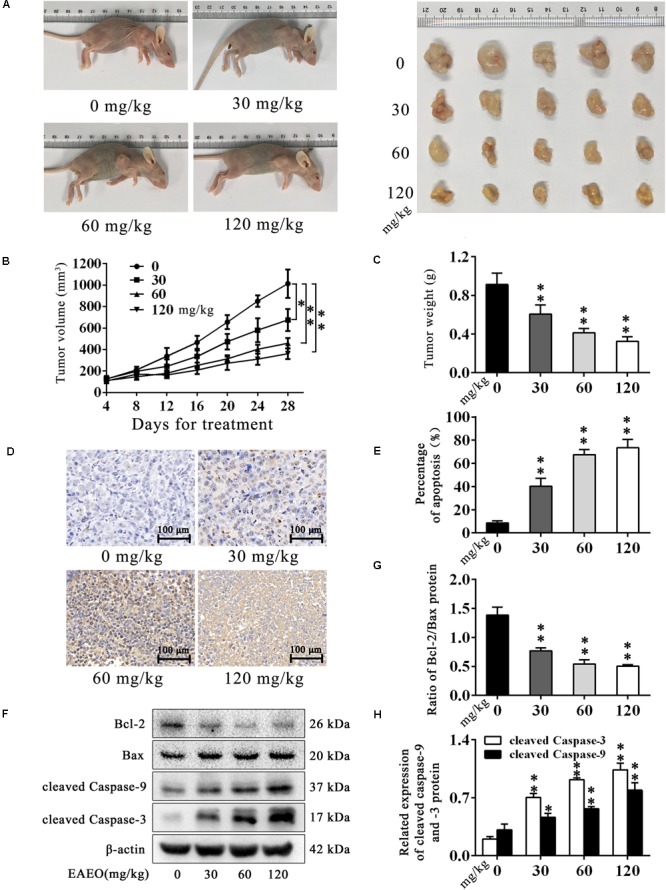
EAEO inhibits the tumor growth and induces apoptosis *in vivo*. **(A,C)** At the end of the experiment, xenografts were dissected out and measure weights. **(B)** Tumor growth curve. Tumor volume was measured every 4 days after implantation. **(D,E)** Tumor sections were prepared and subjected to TUNEL assay. Photographs were taken at the magnification of 200×. The apoptotic nucleus in tumor sections were blinded, quantified and subjected to statistical analysis. **(F)** Xenografts lysates were processed for western blot analysis and probed with the indicated antibodies. **(G)** Ratio of Bcl-2/Bax protein. **(H)** Relative expression of cleaved caspase-9 and -3 protein. Statistical difference was analyzed by ANOVA, ^∗^*P* < 0.05, ^∗∗^*P* < 0.01 compare to control (0 mg/kg served as control).

HepG2-bearing nude mouse model was employed to evaluate anti-HCC effects of EAEO *in vivo*. We found that EAEO treatment resulted in growth inhibition of HepG2 xenografts in mice (**Figures 7A,B**) and decreased tumor weights in a dose-dependent manner (**Figure 7C**). Considering the significant pro-apoptotic activity of EAEO against HCC cells *in vitro*, we next performed TUNEL assays and measured the expression of apoptosis regulatory proteins of tumor to determine whether EAEO induced HepG2 cell apoptosis *in vivo* (Supplementary Figure S2). The results showed that EAEO treatment significantly enhanced apoptotic nucleus in the tumor sections (**Figures 7D,E**), suppressed the ratio of Bcl-2/Bax and increased the expression of cleaved caspase-9 and -3 (**Figures 7E–G**). Together, these results show that EAEO treatment inhibited tumor growth and induced HCC cells apoptosis by mitochondrial apoptotic pathway, further confirming the potential anti-HCC effect of EAEO.

### EAEO Represses ERS-Related Proteins Activity

**FIGURE 8:**
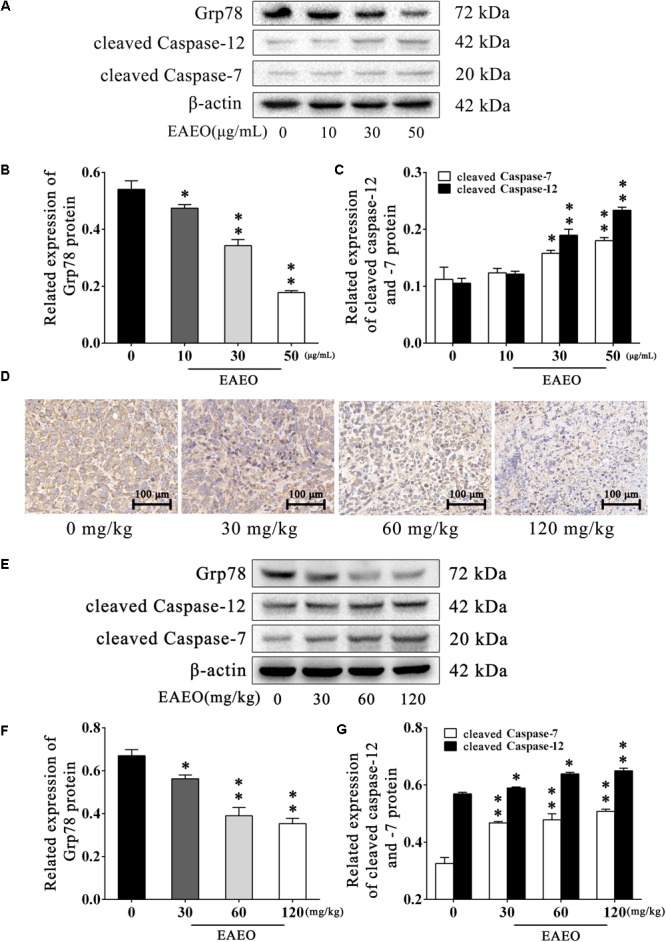
EAEO represses ERS-related proteins activity. **(A)** HepG2 cells were treated with 0, 10, 30, and 50 μg/mL of EAEO for 24 h, after which whole-cell lysates were processed for western blot analysis and probed with the indicated antibodies. **(B)** Related expression of Grp78 protein *in vitro*. **(C)** Relative expression of cleaved caspase-12 and -7 protein *in vitro*. **(D)** The paraffin-embedded xenografts were subjected to IHC for Grp78. Photographs were taken at the magnification of 200×. **(E)** Xenograft lysates were processed for western blot analysis and probed with the indicated antibodies. **(F)** Ratio of Bcl-2/Bax protein *in vivo*. **(G)** Relative expression of cleaved caspase-9 and -3 protein *in vivo*. Statistical difference was analyzed by ANOVA, ^∗^*P* < 0.05, ^∗∗^*P* < 0.01 compare to control (0 μg/mL or 0 mg/kg served as control).

Persistence of ER stress (ERS) induces apoptosis (Szegezdi et al., 2006). Caspase-12 plays a pivotal role in ERS-induced apoptosis, and was mediated by translocation and cleaving of caspase-7 (Nakagawa et al., 2000). On the contrary, glucose-regulated protein (Grp) 78 inhibits ERS-induced apoptosis by blocking caspase activation (Rao et al., 2002). Therefore, we investigated the expression of Grp78, cleaved caspase-12 and -7 via western blot analysis (**Figure 8A**). The results indicated that EAEO down-regulated expression of Grp78 (**Figure 8B**) and increased the expression of cleaved caspase-12 and -7 in a dose-dependent manner (**Figure 8C**). To further confirm the effect of EAEO on ERS-induced apoptosis, we analysis the above proteins of xenografts (**Figure 8E**). As expected, EAEO-treatment declined the expression of Grp78 (**Figure 8F**) and increased the expressions of cleaved caspase-12 and -7 *in vivo* (**Figure 8G**). The observations by immunohistochemical examinations (IHC) consistently confirmed the inhibition of EAEO on Grp78 (**Figure 8D**). These results further showed that EAEO-induced apoptosis was also implemented by the apoptotic signaling with ER (Supplementary Figures S1, S2).

### EAEO Represses STAT3 and AKT Activity

**FIGURE 9:**
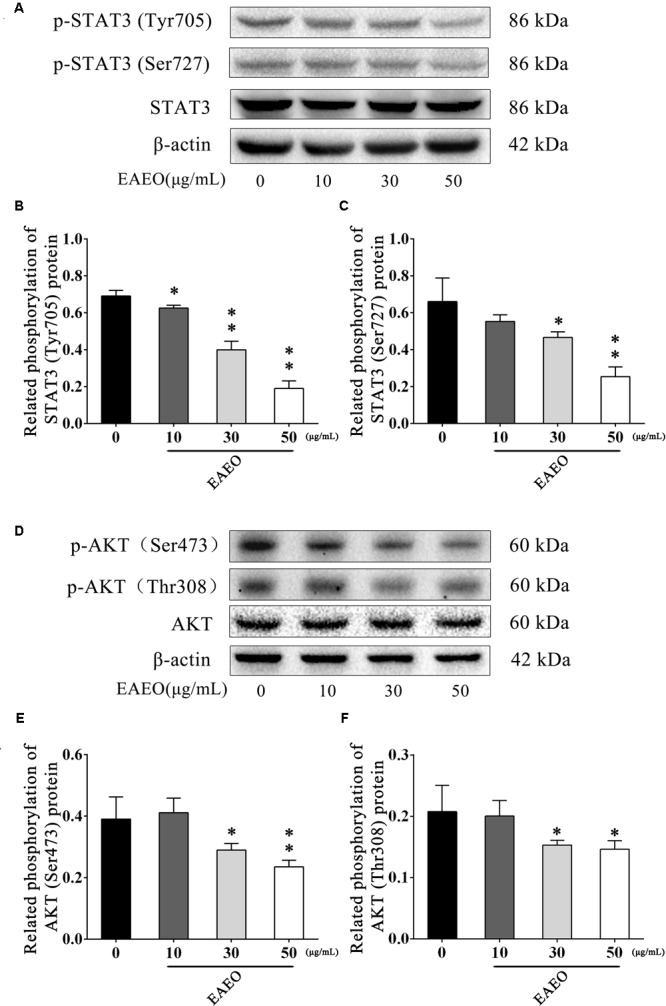
EAEO represses STAT3 and AKT activity *in vitro*. **(A,D)** HepG2 cells were treated with 0, 10, 30, and 50 μg/mL of EAEO for 24 h, after which whole-cell lysates were processed for western blot analysis and probed with the indicated antibodies. **(B)** Related phosphorylation of STAT3 (Tyr705) protein. **(C)** Relative phosphorylation of STAT3 (Ser727) protein. **(E)** Related phosphorylation of AKT (Ser473) protein. **(F)** Related phosphorylation of AKT (Thr308) protein. Statistically difference was analyzed by ANOVA, ^∗^*P* < 0.05, ^∗∗^*P* < 0.01 compare to control (0 μg/mL served as control).

STAT3 is an important transcription factor that regulates the processes of proliferation and apoptosis, and persistently activates in a high number of human cancers (Yu and Jove, 2004). Moreover, Bcl-2 is a typical STAT3-responsive gene (Avalle et al., 2012). Given the down-regulation of Bcl-2 protein in above analysis, we aimed to evaluate changes in STAT3 protein expression in EAEO-induced apoptosis in HCC cells. It has been generally believed that phosphorylated STAT3 as an active form is principally mediated by the phosphorylation at Tyr705 and Ser727 (Levy and Darnell, 2002); hence, the expressions of these two residues were investigated (**Figure 9A**). As shown in **Figures 9B,C**, EAEO dose-dependently repressed the basal phosphorylation of STAT3 at Tyr705 and Ser727 but did not inhibit protein levels of total STAT3.

**FIGURE 10:**
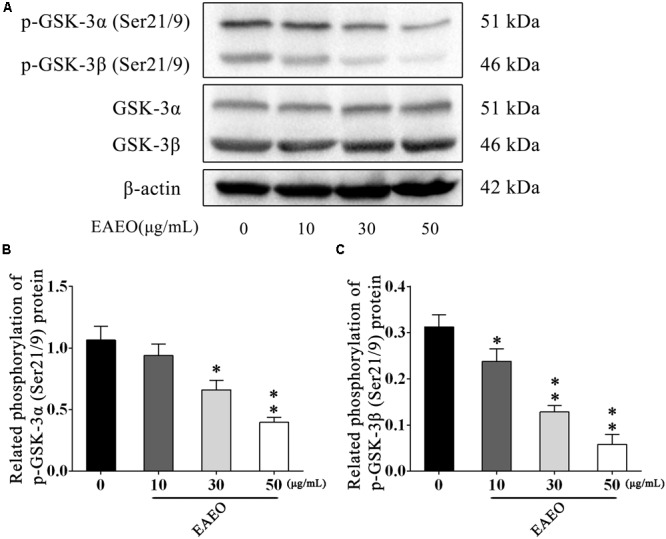
EAEO repressed phosphorylation of GSK-3α/β *in vitro.* **(A)** HepG2 cells were treated with 0, 10, 30, and 50 μg/mL of EAEO for 24 h, after which whole-cell lysates were processed for western blot analysis and probed with the indicated antibodies. **(B)** Related phosphorylation of p-GSK-3α (Ser21/9) protein. **(C)** Relative phosphorylation of p-GSK-3β (Ser21/9) protein. Statistically difference was analyzed by ANOVA, ^∗^*P* < 0.05, ^∗∗^*P* < 0.01 compare to control (0 μg/mL served as control).

The AKT protein as a vital component of PI3K-AKT pathway is another important regulator of cellular proliferation and survival, whose dysregulation is associated with the development of cancer (Vivanco and Sawyers, 2002). Phosphorylation at Ser473 and Thr308 is essential for activation of AKT; thus, the expressions of the two residues were evaluated by western blot (**Figure 9D**). As shown in **Figures 9E,F**, EAEO dose-dependently repressed the phosphorylation of AKT at Ser473 and Thr308 but did not inhibit protein levels of total AKT. GSK-3 is a critical downstream element of the AKT cell survival pathway whose activity can be inhibited by Akt-mediated phosphorylation at Ser21 of GSK-3α and Ser9 of GSK-3β. Inhibiting GSK-3 may improve the expression of tumorigenic proteins like, β-catenin and Cyclin D1. Interestingly, we notice the down-regulation of Cycline D1 which may be related to GSK-3β status. As expected, we observed that EAEO dose-dependently repressed the phosphorylation of GSK-3α/β without inhibiting protein levels of total GSK-3α/β (**Figure 10**). Our data further strengthen the evidence that AKT appears to be a promising target of EAEO using in HCC treatment.

**FIGURE 11:**
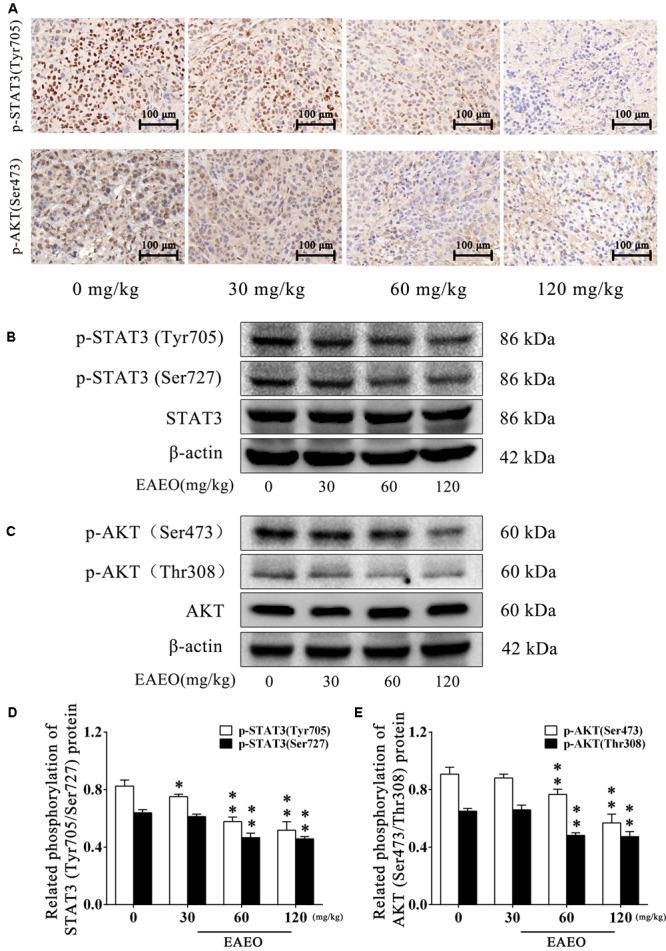
EAEO repressed STAT3 and AKT activity *in vivo*. **(A)** The paraffin-embedded xenografts were subjected to IHC for p-STAT3 (Tyr705) and p-AKT (Ser473). Photographs were taken at the magnification of 200×. **(B,C)** Xenograft lysates were processed for western blot analysis and probed with the indicated antibodies. **(D)** Related phosphorylation of STAT3 (Tyr705/Ser727) protein. **(E)** Related phosphorylation of AKT (Ser473/Thr308) protein. Statistical difference was analyzed by ANOVA, ^∗^*P* < 0.05, ^∗∗^*P* < 0.01 compare to control (0 mg/kg served as control).

To evaluate the effects of EAEO on STAT3 *in vivo*, we measured phosphorylation levels of STAT3 in xenografts. As shown by immunohistochemistry data, phosphorylation levels of STAT3 at Tyr705 in tumor tissues were substantially decreased after EAEO treatment (**Figure 11A**). Consistently, Western blot analysis showed that EAEO dose-dependently suppressed constitutive STAT3 phosphorylation levels at Tyr705 and Ser727 (**Figures 11B,D**). Similarly, we studied the effects of EAEO on AKT *in vivo*. After EAEO treatment, decrease of phosphorylation levels of AKT at Ser473 was observed in IHC assay (**Figure 11A**). Western blot analysis also showed that EAEO dose-dependently suppressed constitutive AKT phosphorylation levels at Ser473 and Thr308 (**Figures 11C,E**). Taken together, these observations suggest that EAEO has the potential to inhibit phosphorylation of STAT3 and AKT *in vivo* (above Immunoblotting bands were provided in Supplementary Figures S1–S3).

## Discussion

The EA is a noxious species for which there is a lack of effective control measures. However, the potential use of EA as a resource in pharmaceuticals industry and agriculture may present an opportunity to counter its notoriety as an environmental hazard. Nevertheless, the pharmacological effects and mechanism of EA on humans remain poorly understood. In our study, EAEO displayed antiproliferative activity against several HCC cells lines (**Figures 2A,B**). Previous experiments showed that the anticancer activity of plant essential oils is mostly due to terpenoids as the major active compounds (Kuttan et al., 2011; Sieniawska et al., 2016; Pudziuvelyte et al., 2017). Analyses of chemical compositions of EAEO by GC-MS revealed that the EAEO is mainly composed of sesquiterpenes, which were probably responsible for the anticancer activity (**Figure 1** and **Table 1**). Our studies unveiled that EAEO dose- and time-dependently inhibited HCC cell proliferation, without inducing cytotoxicity in normal liver cells (**Figures 2C–E**). This hypotoxicity of EAEO is considered a basic requirement for potential chemotherapeutic agents. Herein, we conducted several *in vitro* and *in vivo* studies to further elucidate the potential of EAEO in anti-HCC activity.

The typical pathological hallmarks of HCC cells, like most malignant tumor cells, are activating invasion and metastasis, uncontrolled cell cycle, resistance to apoptosis (Hanahan and Weinberg, 2000; Igney and Krammer, 2002). Often, the prognosis of HCC patients is very poor is generally due to the metastatic spread of cancer cells. Invasiveness and migration ability largely determine the metastatic potential of most solid malignancies including HCC (Chambers et al., 2002). Here, our study showed that EAEO treatment inhibited HepG2 cells migration even in a lower concentration (**Figure 3**). These data suggest that EAEO has inhibitory potential of metastatic spread of cancer cells. Uncontrolled cell cycle is a fundamental aspect of cancer. Normal cells monitor their developmental or other mitogenic signals and decide whether to proliferate, or remain quiescent, whereas cancer cells must evade this control of proliferation in order to survive. This suggests that cancer cells have defects in regulation of the proliferation signals (Nakayama and Nakayama, 2006). Cell cycle checkpoints are crucial control mechanisms in proliferation. Mutations in checkpoint pathways can induce genomic abnormalities possible for survival or continued growth, thereby more likely causing malignant transformation (Massague, 2004). Cell cycle arrest is referenced as a part of a treatment strategy for slowing the progression of cancers. Our FACS data and western blot analysis showed that EAEO induced G_1_ phase arrest and the blockage effects on cell cycle might prevent HCC cells from entering M phase (**Figure 4**).

Apoptosis is an intrinsic self-destruction mechanism linked to many biological processes. Defects in apoptosis mechanisms can disrupt the delicate balance of cell process and might eventually result in cancer (Igney and Krammer, 2002). Even so, cancer cells are destined to die, and have to be under the survival stress from self-defect. Some cancers including HCC are highly dependent on aberrations of the apoptosis signaling pathways to survive, but parts of apoptotic mechanisms may still be preserved. Therefore, restoring apoptosis might be effective against cancers. In fact, various targeted drugs developed from extensive molecular understanding of apoptosis have been designed to kill cancer cells through suppressing key molecules in mutation of apoptosis signaling pathways or enhancing the activation of conservative apoptotic mechanisms (Fesik, 2005; Lopez and Tait, 2015).

Mitochondria play a central role in apoptotic cell death. The relative expression of Bcl-2 and Bax and the activation of caspase-9 and -3 are classical events mediating mitochondrial apoptosis signaling pathway, while Bcl-2 and Bax control mitochondrial permeability. Changing of ratio of Bcl-2/Bax protein leads to *cytochrome C* release, which is associated with the activation of caspase-9 and -3, and eventually induces apoptosis (Masuelli et al., 2017). Our FACS analysis showed the increase of sub G_1_ phase as the sign of nuclear debris from apoptotic cells (**Figure 4**), which was consistent with the apoptotic change of cellular morphology observed under phase contrast microscopy (**Figure 5**). Based on its favorable *in vitro* pro-apoptotic activity against HCC cells, we employed HepG2-bearing nude mouse model and analyzed the expression of apoptosis regulatory proteins. We unveiled that EAEO inhibited the transplanted tumor growth. Besides, the impacts on body weight and organ index of nude mouse were provided in Supplementary Data Sheet S1. Western blot analysis of both *in vitro* and *in vivo* samples showed that the ratio of Bcl-2/Bax was dose-dependently decreased and the expressions of cleaved caspase-9 and -3 were dose-dependently increased after EAEO treatment. These data indicated that EAEO might be a potential anti-HCC agent by activating the mitochondrial apoptosis signaling pathway (**Figures 6, 7**).

Recent studies have also pointed out the fact that the signaling pathway of apoptosis mediated by ERS is a new mechanism of apoptosis. ERS is triggered by the disequilibrium of calcium homeostasis and the accumulation of unfolded or misfolded proteins in ER. Persistence of ERS induces normal cells apoptosis, but in incipient ERS, cells evoke a repair mechanism called unfolded protein response (UPR) to reestablish ER homeostasis in an attempt to stay alive. Many cancers face metabolic dysregulations such as hypoxia, nutrient deprivation, and oxidative stress, resulting in persistent ERS. However, cancer cells seem to benefit from UPR to escape from apoptosis and adapt to the transformed state (Wang et al., 2010). Considering that upregulation of ERS was also detected in HCC and associated with the HCC development (Nakagawa et al., 2014), we examined the impact of EAEO on ERS in HCC. We detected the expression of Grp78 in EAEO-treated HCC cells, whose elevated expression usually correlates with a variety of cancer microenvironmental stresses and initiation of UPR. EAEO dose-dependently suppressed the expression of Grp78 (**Figure 8B**) confirming that EAEO produced an effect on the ERS pathway, which piqued our interest in the involvement of ERS pathway in EAEO-induced HCC cells apoptosis. Caspase-12 as a core molecule, whose activation is mediated by Grp78 and caspas-7, is only implicated in the apoptosis signaling pathway with ERS (Nakagawa et al., 2000). The expressions of caspase-12 and -7 were detected, and the results showed that EAEO treatment increased their activity (**Figure 8C**). These findings were replicated in *in vivo* studies (**Figure 9**). These findings suggested that separate from mitochondrial apoptosis, EAEO might also relieve the inhibition of Grp78 in caspase-12 and -7 to restore apoptosis signaling pathway with ERS.

Normally, the mechanism of apoptosis is also limited by anti-apoptotic pathways. In the present study, we evaluated the expressions of Bcl-2 and Bax, which are associated with STAT3 or AKT signaling. STAT3 and AKT are key molecules constituting significant anti-apoptotic pathways in many cancers (Choudhari et al., 2007). To explore whether STAT3 and AKT signaling were possible mediators of HCC cell apoptosis triggered by EAEO, the activity of STAT3 and AKT were detected both *in vitro* and *in vivo*. The results confirmed our conjecture that EAEO treatment inhibited the activity of STAT3 and AKT in HCC (**Figures 9–11**), suggesting that EAEO-induced HCC cell apoptosis might be triggered by the inhibition of STAT3 and AKT.

**FIGURE 12:**
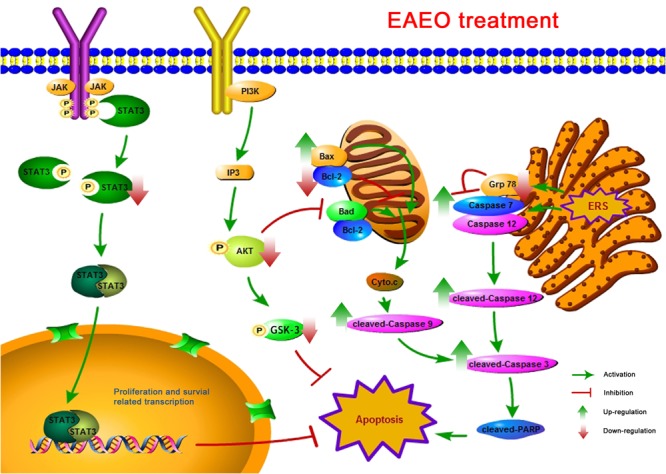
A schematic summary for the mechanisms of EAEO-induced HCC cells apoptosis in the present study.

In summary, analyses of chemical compositions revealed that the EAEO is mainly composed of sesquiterpenes, and the dominant sesquiterpenes are torreyol, aristolone, and α-bisabolol, followed by α-curcumene, β-bisabolene, and β-sesquiphellandrene. Our pharmacological study indicated that EAEO was capable of arresting HCC cells cycle in G1 phase, inhibit cell proliferation and migration, but showed little toxic in normal liver cells. Animal experiments further verified that EAEO induced cell apoptosis. The molecular mechanism of EAEO-induced HCC cell apoptosis was implemented by the activation of apoptosis signaling pathway in the mitochondria and ER, as well as repressing the phosphorylation of STAT3 and AKT (**Figure 12**). Our present findings suggest EAEO has the potential to be a STAT3 and AKT inhibitor in targeted chemotherapeutic drug development.

## Ethics Statement

This study was carried out in accordance with the recommendations of ‘Guidelines for Tumor Induction in Mice and Rats, Animal Care and Use Committee of South-Central University for Nationalities.’ The protocol was approved by the ‘Animal Care and Use Committee of South-Central University for Nationalities.’

## Author Contributions

HC conceived the study, performed *in vivo* pharmacological activities (Cell culture, MTT assay, Hoesct 33258 staining and FACS), and carried out data analysis and wrote the manuscript. BZ performed the western blotting analysis. JY and YH run the *in vivo* pharmacological activities (nude mouse model, TUNEL and IHC). XM, SD, and YW prepared plant material and performed GC-MS analysis. XY and JqY supervised all work.

## Conflict of Interest Statement

The authors declare that the research was conducted in the absence of any commercial or financial relationships that could be construed as a potential conflict of interest.
