# Supplementary material for: Essential Oil Derived From Eupatorium adenophorum Spreng. Mediates Anticancer Effect by Inhibiting STAT3 and AKT Activation to Induce Apoptosis in Hepatocellular Carcinoma
Source: Front Pharmacol. 2018 May 15;9:483. doi: 10.3389/fphar.2018.00483 (PMC5963395; doi:10.3389/fphar.2018.00483)
Supplement: Supplementary file 1 [file Data_sheet_1.DOCX]

Supplementary materials

**The body weight and organ index of HepG2-bearing nude mouse model**

1. **Methods**

HepG2-bearing nude mouse models were randomized in 4 groups (n = 5 mice per group) and treated i.p. with EAEO (0, 30, 60, 120 mg/kg/2 days) for about 3 weeks. Body weight was measured every 2 days. Three weeks later, mice were sacrificed, the transplanted corresponding organs were dissected out and weighed. Organ index was calculated by the formula: Organ index=organ weight (g) / body weight (g) × 100.

2. **Results**

The spleen are essential immune organs, and liver and renal is vulnerable to chemotherapy drug. We observed distinct splenomegaly in HepG2-bearing nude mouse model. After measurement, we found that EAEO treatment reduced the spleen index without effecting the liver index and renal index (TABLE 1). However, we also observed the slightly weight loss in the treatment group (FIGURE 1). These observations implied that toxic effects of EAEO on the vital organs of tumor-bearing mice are undetectable in our system, but there is a risk of weight loss.

**
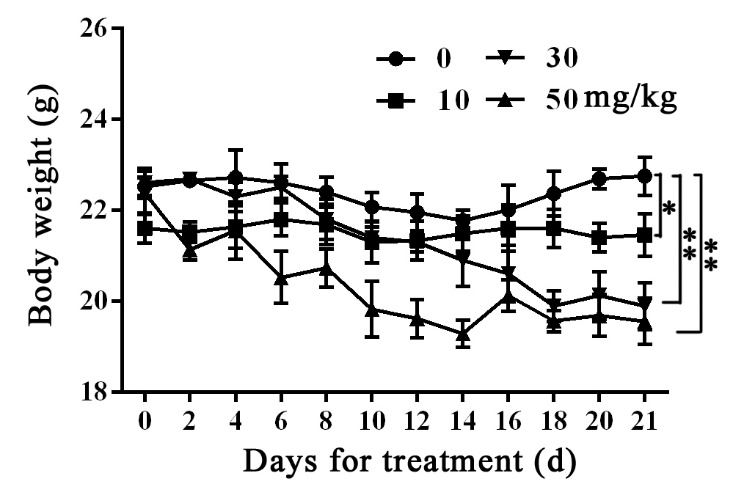
**

**FIGURE 1** Effect of EAEO on HepG2-bearing nude mouse models body weight

**TABLE 1 Effect of EAEO on organ indexes in tumor-bearing mice. (mean ± SD, n = 5)**

| Dose (mg/kg) | Organ index | | |
| --- | --- | --- | --- |
|  | Spleen index | Liver index | Renal index |
| 0 | 3.11 ± 0.032 | 7.73 ± 0.089 | 1.74 ± 0.021 |
| 10 | 2.99 ± 0.025 | 7.38 ± 0.075 | 1.74 ± 0.019 |
| 30 | 2.45 ± 0.029 | 7.52 ± 0.082 | 1.76 ± 0.022 |
| 50 | 2.23 ± 0.031 | 7.72 ± 0.084 | 1.75 ± 0.027 |
