# Supplementary figures and images for: Essential Oil Derived From Eupatorium adenophorum Spreng. Mediates Anticancer Effect by Inhibiting STAT3 and AKT Activation to Induce Apoptosis in Hepatocellular Carcinoma
Source: Front Pharmacol. 2018 May 15;9:483. doi: 10.3389/fphar.2018.00483 (PMC5963395; doi:10.3389/fphar.2018.00483)

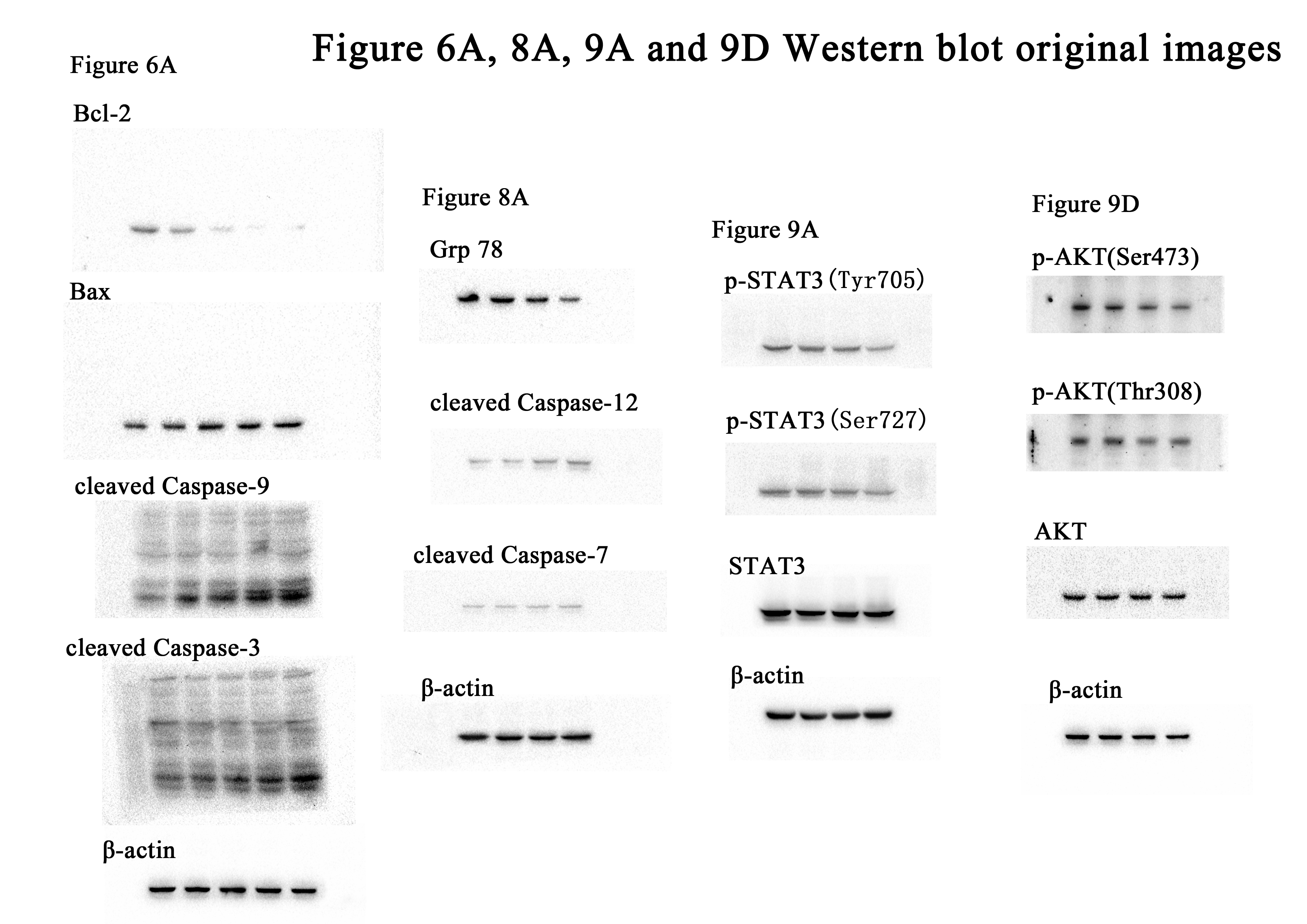

Supplement: Supplementary file 2 [file Image_1.jpg]

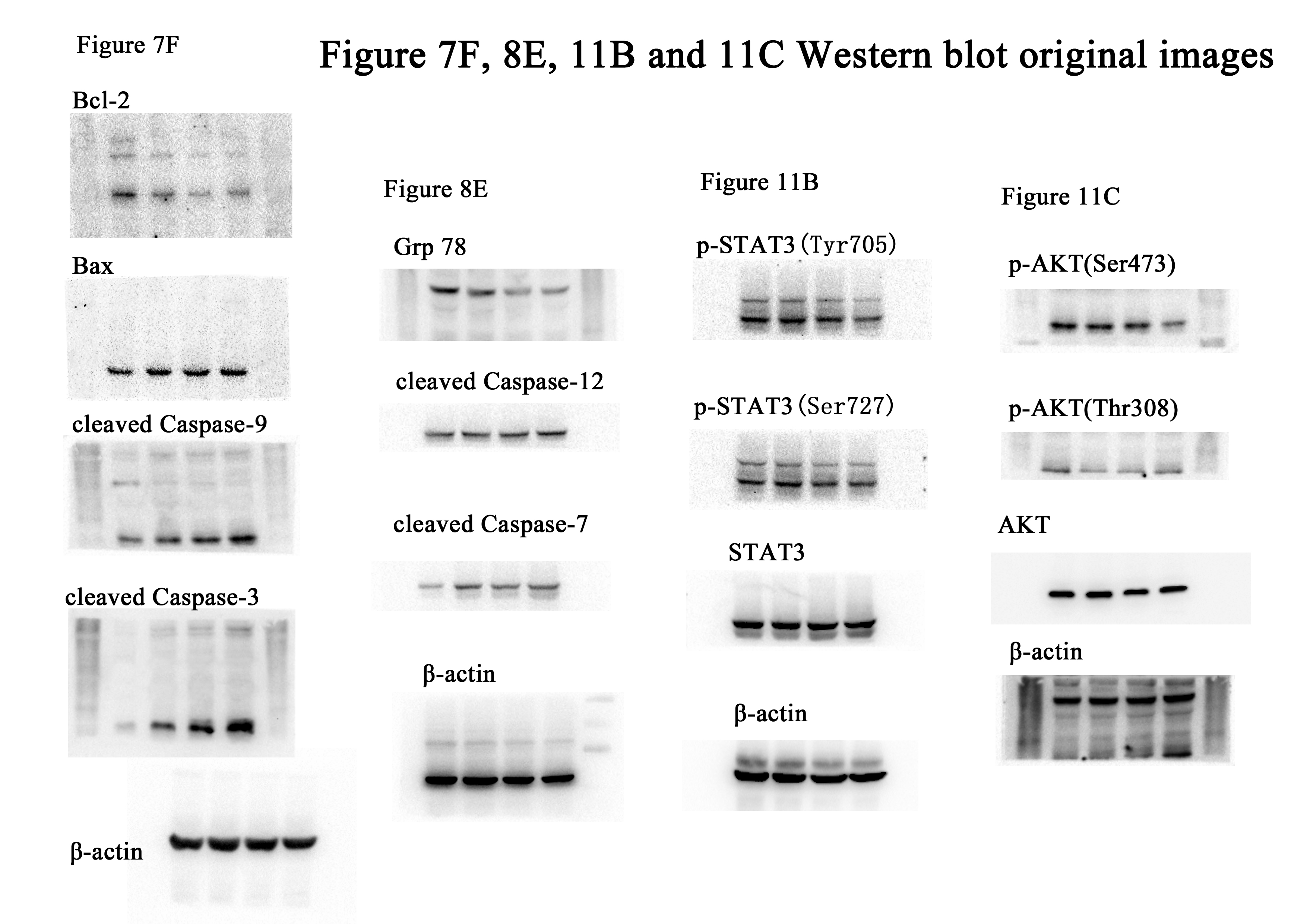

Supplement: Supplementary file 3 [file Image_2.jpg]

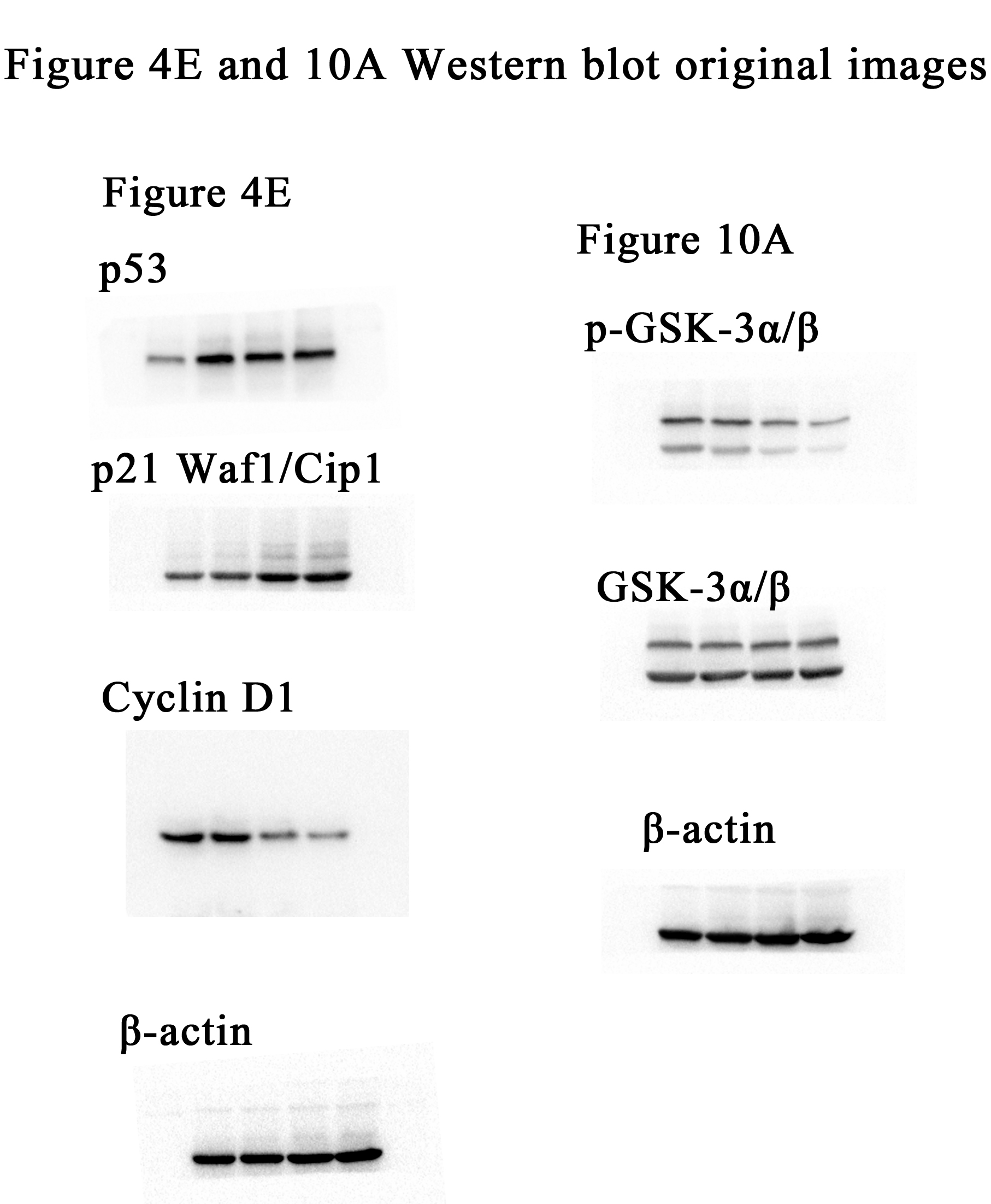

Supplement: Supplementary file 4 [file Image_3.jpg]
